# Supplementary material for: Distinct YFV Lineages Co-circulated in the Central-Western and Southeastern Brazilian Regions From 2015 to 2018
Source: Front Microbiol. 2019 May 24;10:1079. doi: 10.3389/fmicb.2019.01079 (PMC6543907; doi:10.3389/fmicb.2019.01079)
Supplement: Supplementary file 1 [file Data_Sheet_1.PDF]

Supplementary information of "**Distinct YFV lineages co-circulated in the Central-Western and Southeastern Brazilian regions from 2015 to 2018**"

Edson Delatorre[1+], Filipe Vieira Santos de Abreu[2,3+], Ieda Pereira Ribeiro[4+], Mariela Martínez Gómez[4,5], Alexandre Araújo Cunha dos Santos[4], Anielly Ferreira-de-Brito[2], Maycon Sebastião Alberto Santos Neves[2], Iule Bonelly[2], Rafaella Moraes de Miranda[2], Nathália Dias Furtado[4], Lidiane Menezes Souza Raphael[4], Lucileis de Fátima Fernandes da Silva[6], Márcia Gonçalves de Castro[2], Daniel Garkauskas Ramos[7], Alessandro Pecego Martins Romano[7], Esper Georges Kallás[8], Ana Carolina Paulo Vicente[1], Gonzalo Bello[9§], Ricardo Lourenço-de-Oliveira[2§], Myrna Cristina Bonaldo[4§]

[1] Laboratório de Genética Molecular de Microorganismos, Instituto Oswaldo Cruz, Fundação Oswaldo Cruz, Rio de Janeiro, Brazil
[2] Laboratório de Mosquitos Transmissores de Hematozoários, Instituto Oswaldo Cruz, Fundação Oswaldo Cruz, Rio de Janeiro, Brazil
[3] Instituto Federal do Norte de Minas Gerais, Salinas, MG, Brazil
[4] Laboratório de Biologia Molecular de Flavivírus, Instituto Oswaldo Cruz, Fundação Oswaldo Cruz, Rio de Janeiro, Brazil
[5] División Biología Molecular y Genética, Departamento de Biología Molecular, Instituto de Investigaciones Biológicas Clemente Estable, Montevideo, Uruguay
[6] Laboratório Central de Saúde Pública Dr. Giovanni Cysneiros, Secretaria de Saúde de Goiás, Brazil
[7] Coordenação Geral de Vigilância das Doenças Transmissíveis, Departamento de Vigilância das Doenças Transmissíveis, Secretaria de Vigilância em Saúde, Ministério da Saúde, Brasília, DF, Brazil,
[8] Departamento de Moléstias Infecciosas, Hospital das Clinicas HCFMUSP, Faculdade de Medicina, Universidade de São Paulo, Brazil.
[9] Laboratório de AIDS e Imunologia Molecular, Instituto Oswaldo Cruz, Fundação Oswaldo Cruz, Rio de Janeiro, Brazil

Correspondence to:
Edson Delatorre
edsonod@ioc.fiocruz.br/delatorre.ioc@gmail.com

Supplementary Table 1. Information about all YFV complete genomes used in this study.

Accession number	Country	State	Collection date	Host	Analysis*	Lat	Long
MH018099	Brazil	Bahia	2017-03-10	Monkey	ML/B/P	-15,0378010	-41,9346810
MF170972	Brazil	Espírito Santo	2017-03-31	Human	ML/B/P	-20,2632020	-40,4165490
MF170974	Brazil	Espírito Santo	2017-03-29	Human	ML/B/P	-20,2632020	-40,4165490
MF170978	Brazil	Espírito Santo	2017-03-28	Human	ML/B/P	-20,0252960	-40,7439310
MF170976	Brazil	Espírito Santo	2017-01-25	Human	ML/B/P	-20,2261658	-41,6886832
MF170980	Brazil	Espírito Santo	2017-03-23	Human	ML/B/P	-20,6356507	-40,7484424
MF423374	Brazil	Espírito Santo	2017-02-23	Mosquito	ML/B/P	-20,2855556	-40,8375000
KY885001	Brazil	Espírito Santo	2017-02-22	Monkey	ML/B/P	-20,2855556	-40,8375000
MF170970	Brazil	Espírito Santo	2017-03-21	Human	ML/B/P	-20,3646483	-40,6585797
MF423373	Brazil	Espírito Santo	2017-02-21	Mosquito	ML/B/P	-20,2855556	-40,8375000
KY885000	Brazil	Espírito Santo	2017-02-20	Monkey	ML/B/P	-20,2855556	-40,8375000
MF170968	Brazil	Espírito Santo	2017-04-13	Human	ML/B/P	-20,4124966	-40,6749858
MF170973	Brazil	Espírito Santo	2017-04-10	Human	ML/B/P	-20,3646483	-40,6585797
MF170977	Brazil	Espírito Santo	2017-02-10	Human	ML/B/P	-20,3601311	-41,2470343
MF170979	Brazil	Espírito Santo	2017-04-06	Human	ML/B/P	-20,0252960	-40,7439310
MF170981	Brazil	Espírito Santo	2017-04-05	Human	ML/B/P	-20,0987046	-40,5269434
MF170975	Brazil	Espírito Santo	2017-03-03	Human	ML/B/P	-20,1475033	-41,2868561
JF912179	Brazil	Goiás	1980	Mosquito	ML/B	-	-
JF912187	Brazil	Goiás	2000	Human	ML/B	-	-
JF912188	Brazil	Goiás	2000	Human	ML/B	-	-
JF912185	Brazil	Mato Grosso do Sul	1992	Mosquito	ML/B	-	-
MH018101	Brazil	Minas Gerais	2003-08-15	Human	ML/B	-	-
MH018100	Brazil	Minas Gerais	2003-03-15	Human	ML/B	-	-
MF170971	Brazil	Minas Gerais	2017-01-30	Monkey	ML/B/P	-20,2452770	-46,3658330
MH018091	Brazil	Minas Gerais	2017-01-30	Human	ML/B/P	-17,4328950	-41,9989720
MH018079	Brazil	Minas Gerais	2017-01-28	Human	ML/B/P	-18,0340920	-41,6833370
MH018080	Brazil	Minas Gerais	2017-01-28	Human	ML/B/P	-17,5981970	-41,8004410
MH018092	Brazil	Minas Gerais	2017-01-27	Human	ML/B/P	-18,0340920	-41,6833370
MH484427	Brazil	Minas Gerais	2017-01-24	Monkey	ML/B/P	-18,0779540	-43,2478260
JF912186	Brazil	Minas Gerais	1994	Human	ML/B		
MH484434	Brazil	Minas Gerais	2017-01-22	Human	ML/B/P	-18,4968390	-42,7979660
MH018088	Brazil	Minas Gerais	2017-01-21	Human	ML/B/P	-17,8600090	-41,5091040
MH018082	Brazil	Minas Gerais	2017-02-20	Monkey	ML/B/P	-20,3589040	-47,2356990
MH018093	Brazil	Minas Gerais	2017-01-20	Human	ML/B/P	-19,9748220	-42,1375090
MH018076	Brazil	Minas Gerais	2017-01-19	Human	ML/B/P	-17,6095930	-42,1344660
MH018095	Brazil	Minas Gerais	2017-01-19	Monkey	ML/B/P	-18,6658770	-43,0816280
MH018078	Brazil	Minas Gerais	2017-01-18	Human	ML/B/P	-17,4328950	-41,9989720
MH018083	Brazil	Minas Gerais	2017-02-17	Monkey	ML/B/P	-20,3001340	-42,4747050

MF465805	Brazil	Minas Gerais	2017-01-17	Human	ML/B/P	- 15,4878000	- 44,3619000
MH018064	Brazil	Minas Gerais	2017-02-15	Monkey	ML/B/P	- 22,0195820	- 46,2609600
MH018066	Brazil	Minas Gerais	2017-02-15	Monkey	ML/B/P	- 22,2668370	- 46,3755990
MH018065	Brazil	Minas Gerais	2017-02-14	Monkey	ML/B/P	- 20,3223950	- 46,7865520
MH018067	Brazil	Minas Gerais	2017-02-13	Monkey	ML/B/P	- 21,8710940	- 46,4008720
MH018096	Brazil	Minas Gerais	2017-01-13	Monkey	ML/B/P	- 18,2213480	- 42,4985130
MH018090	Brazil	Minas Gerais	2017-01-12	Human	ML/B/P	- 19,7624160	- 42,0461860
MH018084	Brazil	Minas Gerais	2017-02-09	Monkey	ML/B/P	- 17,5981970	- 41,8004410
MH484430	Brazil	Minas Gerais	2017-02-09	Monkey	ML/B/P	- 19,8502340	- 47,3706550
MH484429	Brazil	Minas Gerais	2017-02-03	Monkey	ML/B/P	- 15,5694080	- 45,3745360
MH018089	Brazil	Minas Gerais	2017-01-02	Human	ML/B/P	- 17,5981970	- 41,8004410
JF912180	Brazil	Pará	1981	Human	ML/B	-	-
JF912182	Brazil	Pará	1984	Human	ML/B	-	-
JF912183	Brazil	Pará	1984	Human	ML/B	-	-
JF912184	Brazil	Pará	1987	Human	ML/B	-	-
MF434851	Brazil	Rio de Janeiro	2017-04-25	Human	ML/B/P	- 22,4619444	- 42,3080556
MF538784	Brazil	Rio de Janeiro	2017-02-26	Human	ML/B/P	- 20,8216667	- 41,9108333
MF538785	Brazil	Rio de Janeiro	2017-04-21	Monkey	ML/B/P	- 22,3975000	- 43,1825000
MF423377	Brazil	Rio de Janeiro	2017-04-19	Monkey	ML/B/P	- 21,8847222	- 42,5416667
MF423378	Brazil	Rio de Janeiro	2017-04-19	Monkey	ML/B/P	- 21,8847222	- 42,5416667
MF538783	Brazil	Rio de Janeiro	2017-03-18	Human	ML/B/P	- 22,4863889	- 42,2016667
MF538782	Brazil	Rio de Janeiro	2017-03-16	Human	ML/B/P	- 21,6380556	- 41,7636111
MF423376	Brazil	Rio de Janeiro	2017-04-13	Monkey	ML/B/P	- 22,2397222	- 42,1513889
MF538786	Brazil	Rio de Janeiro	2017-06-05	Monkey	ML/B/P	- 22,4933333	- 42,9497222
MF423375	Brazil	Rio de Janeiro	2017-04-04	Monkey	ML/B/P	- 22,3088889	- 42,0005556
KY861728	Brazil	Rio Grande do Sul	2008-02-09	Monkey	ML/B	-	-
JF912189	Brazil	Rio Grande do Sul	2001	Mosquito	ML/B	-	-
JF912190	Brazil	Roraima	2002	Human	ML/B	-	-
HM582851	Trinidad and Tobago	-	2009	Monkey	ML/B	-	-
KM388817	Venezuela	-	2004	Monkey	ML/B	-	-
KM388814	Venezuela	-	2005	Human	ML/B	-	-
KM388818	Venezuela	-	2006	Monkey	ML/B	-	-
KM388815	Venezuela	-	2007	Monkey	ML/B	-	-
KM388816	Venezuela	-	2010	Monkey	ML/B	-	-

* ML/B, maximum likelihood and Bayesian; ML/B/P, ML/B and phylogeography.

Supplementary Table 2. Molecular cock rates and T_{MRCA} obtained for the $YFV_{2015\text{-}2018}$ lineage using different combinations of clock rate priors and phylogeographic models

Dataset	Coalescent model	Model/Prior clock rate	Phylogeographic model	Posterior clock rate (s/s/y)	T_{MRCA} $YFV_{2015\text{-}2018}$
SA-I	Bayesian skyline	UCLD/CTMC	-	4.3×10^{-4} ($3.1 \times 10^{-4} – 5.7 \times 10^{-4}$)	2013.8 (2012.4-2014.9)
Brazil 2015-2018		UCLD/Normal median = $4.5{\times}10^{-4}$ stdev = $1.0{\times}10^{-4}$	discrete symmetric	5.4×10^{-4} ($3.9 \times 10^{-4} – 7.0 \times 10^{-4}$)	2014.3 (2012.8-2015.5)
			discrete asymmetric	5.4×10^{-4} ($3.9 \times 10^{-4} – 6.9 \times 10^{-4}$)	2014.4 (2012.8-2015.5)
		UCLD/Normal median = $4.5{\times}10^{-4}$ stdev = $1.0{\times}10^{-4}$	continuous RRW lognormal	5.4×10^{-4} ($3.7 \times 10^{-4} – 7.0 \times 10^{-4}$)	2014.0 (2012.2-2015.3)

SA-I, South American I genotype; UCLD, uncorrelated lognormal relaxed clock; CTMC, continuous-time Markov chain; RRW, relaxed random walk; s/s/y, substitutions/site/year; TMRCA, time for the most recent common ancestor.

Supplementary Table 3. Amino acid molecular signature present in 2016-2018 YFV samples from Southeastern Brazil.

2016-2018 YFV genomes (GenBank accession number)	Polyprotein position								
	108	1572	1605	2607	2644	2679	2803	3149	3215
KY885000; KY885001; MF170968; MF170970; MF170971; MF170972; MF170973; MF170974; MF170975; MF170976; MF170977; MF170978; MF170979; MF170980; MF170981; MF423373; MF423374; MF423375; MF423376; MF423377; MF423378; MF434851; MF465805; MF538782; MF538783; MF538784; MF538785; MF538786; MH018065; MH018066; MH018067; MH018076; MH018078; MH018079; MH018080; MH018082; MH018083; MH018090; MH018091; MH018092; MH018095; MH018096; MH018099; MH484427; MH484429; MH484430; MH484434; MK333798; MK333799; MK333800; MK333801; MK333802; MK333805; MK333806; MK333807; MK333808; MK333809	I	D	K	R	I	S	S	A	S
MK728873	I	D	K	R	I	S	S	V	S
MH018088; MH018084; MH018089; MH018064	I	D	K	Q	I	S	S	A	S
MH018093	I	E	K	Q	I	S	S	A	S

Supplementary Table 4. Comparison of discrete spatial models fit to the 2015-2018 Brazilian YFV dataset.

**Model**	**PS Log ML**	**Models compared**	**Log BF**	**SS Log ML**	**Models compared**	**Log BF**
Sym	-16286	-	-	-16287	-	-
Asym	-9908	Asym/Sym	6378	-4789	Asym/Sym	11498

Sym, symmetric; Asym, asymmetric, PS, path sampling, SS, stepping-stone; ML, marginal likelihood; BF, Bayes factor.

Supplementary Table 5. Comparison of continuous spatial models fit to the 2015-2018 Brazilian YFV dataset and estimate of $YFV_{2015-2018}$ lineage dispersal rate under the different models.

	**Homogeneous Brownian diffusion**	**Heterogeneous RRW Cauchy**	**Heterogeneous RRW Gamma**	**Heterogeneous RRW Lognormal**
PS - Log ML[a]	-16402	-16379	-16364	-15598
PS – Log BF[b]	804	781	766	
SS - Log ML[a]	-16402	-16379	-16364	-15202
SS – Log BF[b]	1200	1177	1162	
Dispersal rate (km/day)[c]	0.6 (0.4 – 0.8)	0.5 (0.4 – 0.7)	0.6 (0.4- 0.7)	0.5 (0.4 – 0.7)

[a] Log marginal likelihood (ML) estimates for the different continuous phylogeographic models obtained using the path sampling (PS) and stepping-stone sampling (SS) methods. [b] The Log Bayes factor (BF) is the difference of the Log ML between of alternative (H1) and null (H0) models (H1/H0). Log BFs > 3 indicates that model H1 is more strongly supported by the data than model H0. [c] Posterior mean and 95% HPD (in parenthesis) estimates of the dispersal rate.
