# Supplementary material for: Distinct YFV Lineages Co-circulated in the Central-Western and Southeastern Brazilian Regions From 2015 to 2018
Source: Front Microbiol. 2019 May 24;10:1079. doi: 10.3389/fmicb.2019.01079 (PMC6543907; doi:10.3389/fmicb.2019.01079)
Supplement: Supplementary file 2 [file Data_Sheet_2.PDF]

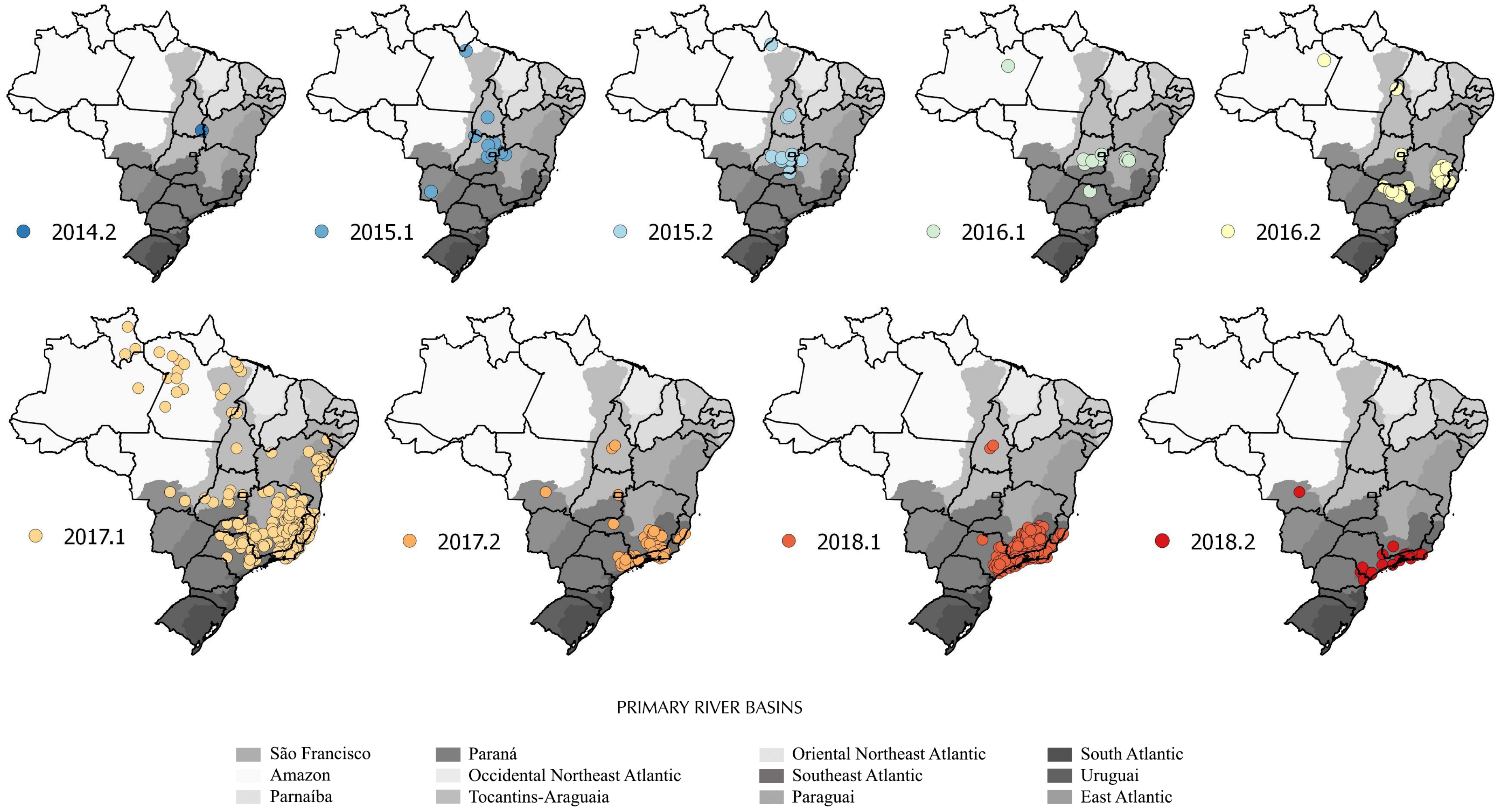

Supplementary Figure 1. Half-yearly based spatial spread of YFV in Brazil from the 2nd semester 2014 (2014.2) to 2nd semester 2018 (2018.2) according to epidemiological records. Dots corresponds to the centroid of municipalities where human and/or non-human YFV infections were laboratory confirmed. The gray-shaded background underlines primary Brazilian river basins; additional details on tributary basins are available from https://mapas.ibge.gov.br/images/pdf/mapas/mappag99.pdf . Source: SVS-Brazilian Ministry of Health.
