# Supplementary material for: Distinct YFV Lineages Co-circulated in the Central-Western and Southeastern Brazilian Regions From 2015 to 2018
Source: Front Microbiol. 2019 May 24;10:1079. doi: 10.3389/fmicb.2019.01079 (PMC6543907; doi:10.3389/fmicb.2019.01079)
Supplement: Supplementary file 3 [file Data_Sheet_3.PDF]

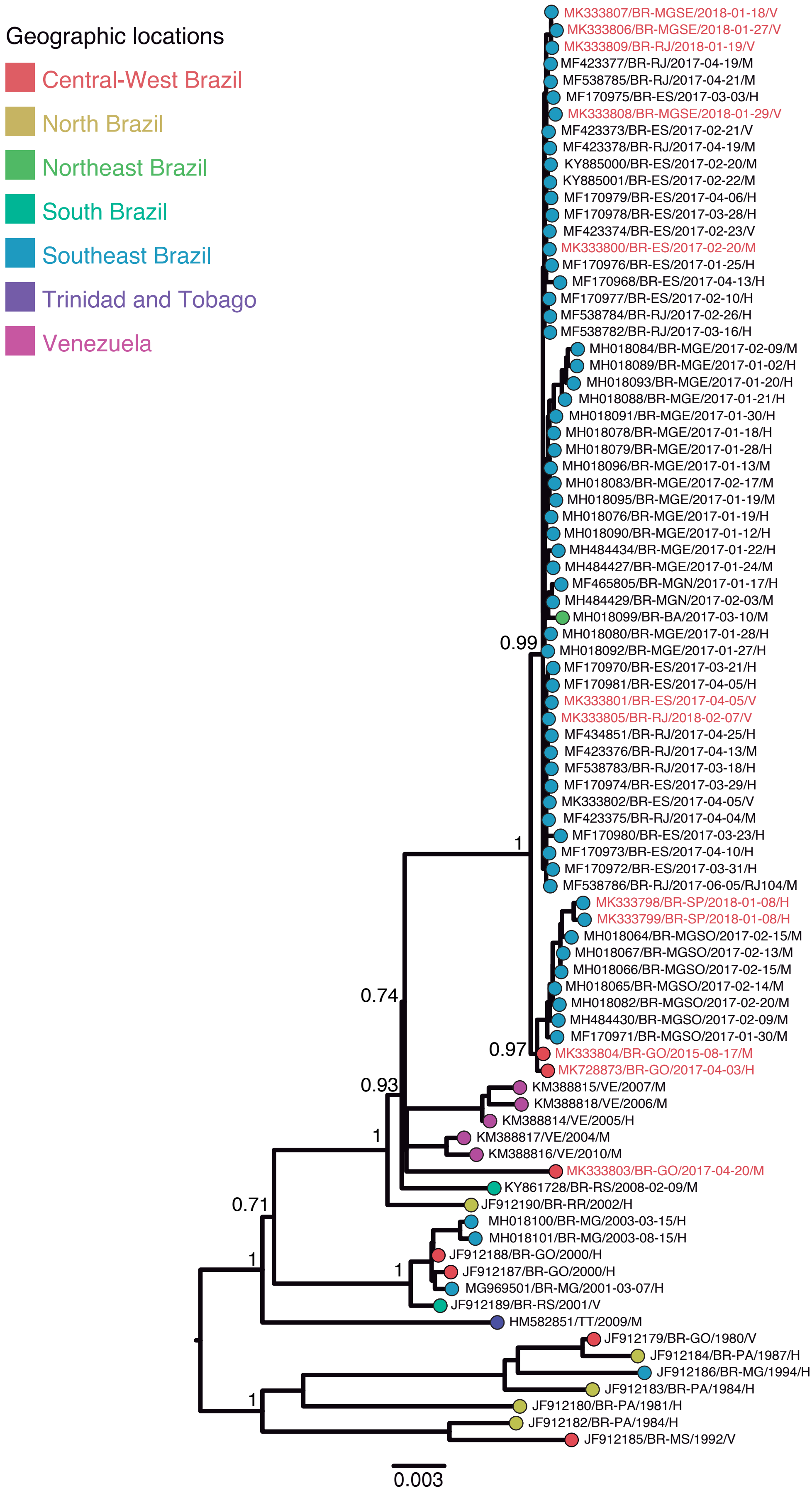

Supplementary Figure 2. Maximum likelihood phylogeny of YFV South American I complete genome sequences. The aLRT support value of key nodes are indicated. Tip circcles are colored following the legend at top left indicating the Brazilian region or country of sampling. The branch lengths are drawn to scale with bar at the bottom indicating nucleotide substitutions per site.
Tips names of the sequences from this study were colored red. The last letter of the sequence name indicate the host: H - human; V - mosquito vector; M - non-human primate.
