# Supplementary material for: Distinct YFV Lineages Co-circulated in the Central-Western and Southeastern Brazilian Regions From 2015 to 2018
Source: Front Microbiol. 2019 May 24;10:1079. doi: 10.3389/fmicb.2019.01079 (PMC6543907; doi:10.3389/fmicb.2019.01079)
Supplement: Supplementary file 4 [file Data_Sheet_4.PDF]

Supplementary Figure 3. Amino acid polymorphisms in YFV in the precursor polyprotein

		Viral proteins	C						prM				E						NS1										NS2A															NS2B							NS3																				
Subclade		YFV sample / polyprotein amino acid position	9	79	82	105	**108**	119	127	128	163	258	405	432	462	483	672	765	787	808	826	881	953	1054	1062	1064	1110	1116	1133	1136	1146	1150	1178	1185	1197	1203	1235	1249	1277	1291	1308	1309	1313	1396	1408	1445	1457	1472	1477	1482	1550	**1572**	1591	**1605**	1646	1666	1669	1679	1701	1811	1834	1835	1927	1938	1979	1992	1998	2006	2010	2045	2049
IE	BR-ES	KY885000/ES504/2017/DomingosMartins/M	K	V	R	V	I	V	R	N	P	A	T	A	R	M	Y	V	G	N	A	P	Y	K	S	V	P	K	I	I	A	V	I	T	N	Y	T	M	V	A	V	L	S	V	R	K	A	L	F	A	K	D	N	K	N	Q	P	I	A	S	N	T	R	S	A	M	G	V	E	T	K
IE	BR-RJ	MK333809/VL2926/2018/Valença/V	.	.	.	.	.	.	.	.	.	.	.	.	.	.	.	.	.	.	.	.	.	.	.	.	.	.	.	.	.	.	.	.	.	.	.	.	.	.	.	.	.	.	.	.	.	.	.	.	.	.	.	.	.	.	.	.	.	.	.	.	.	.	.	.	.	.	.	.	.
IE	BR-MG-SE	MK333808/MG3155/2018/Belmiro Braga/V	.	.	.	.	.	.	.	.	.	.	.	.	.	.	.	.	.	.	.	.	.	.	.	.	.	.	.	.	.	.	.	.	.	.	.	.	.	.	.	.	.	.	.	R	.	.	.	.	.	.	.	.	.	.	.	.	.	.	.	.	.	.	.	.	.	.	.	.	.
IE	BR-MG-SE	MK333807/MG3121/2018/Belmiro Braga/V	.	.	.	.	.	.	.	.	.	.	.	.	.	.	.	.	.	.	.	.	.	.	.	.	.	.	.	.	.	.	.	.	.	.	.	.	.	.	.	.	.	.	.	.	.	.	.	.	.	.	.	.	.	.	.	.	.	.	.	.	.	.	.	.	.	.	.	.	.
IE	BR-MG-SE	MK333806/JF2793/2018/Juiz de Fora/V	.	.	.	.	.	.	.	.	.	.	.	.	.	.	C	.	.	.	.	.	.	.	.	.	.	.	.	.	.	.	.	.	.	.	.	.	.	.	.	.	.	.	.	.	.	.	.	.	.	.	.	.	.	.	.	.	.	.	.	.	.	.	.	.	.	.	.	.	.
IE	BR-RJ	MF423377/2017/RJ95/Carmo/M	.	.	.	.	.	.	.	.	.	.	.	.	.	.	.	.	.	.	.	.	.	.	.	.	.	.	.	.	.	.	.	.	.	.	.	.	.	.	.	.	.	.	.	.	.	.	.	.	.	.	.	.	.	.	.	.	.	.	.	.	.	.	.	.	.	.	.	.	.
IE	BR-RJ	MF423378/2017/RJ96/Carmo/M	.	.	.	.	.	.	.	.	.	.	.	.	.	.	.	.	.	.	.	.	.	.	.	.	.	.	.	.	.	.	.	.	.	.	.	.	.	.	.	.	.	.	.	.	.	.	.	.	.	.	.	.	.	.	.	.	.	.	.	.	.	.	.	.	.	.	.	.	.
IE	BR-RJ	MF538785/2017/RJ97/Petropolis/M	.	.	.	.	.	.	.	.	.	.	.	.	.	.	.	.	.	.	.	.	.	.	.	.	.	.	.	.	.	.	.	.	.	.	.	.	.	.	.	.	.	.	.	.	.	.	.	.	.	.	.	.	.	.	.	.	.	.	.	.	.	.	.	.	.	.	.	.	.
IE	BR-ES	MF423373/2017/PA193/Domingos Martins/V	.	.	.	.	.	.	.	.	.	.	.	.	.	.	.	.	.	.	.	.	.	.	.	.	.	.	.	.	.	.	.	.	.	.	.	.	.	.	.	.	.	.	.	.	.	.	.	.	.	.	.	.	.	.	.	.	.	.	.	.	.	.	.	.	.	.	.	.	.
IE	BR-ES	MF170975/2017/Brejetuba /H	.	.	.	.	.	.	.	.	.	.	.	.	.	.	.	.	.	.	.	.	.	.	.	.	.	.	.	.	.	.	.	.	.	.	.	.	.	.	.	.	.	.	.	.	.	.	.	.	.	.	.	.	.	.	.	.	.	.	.	.	.	.	.	.	.	.	.	.	.
IE	BR-ES	MK333800/ ES03/2017/Domingos Martins/M	.	.	.	.	.	.	.	.	.	.	.	.	.	.	.	.	.	.	.	.	.	.	.	.	.	.	.	.	.	.	.	.	.	.	.	.	.	.	.	.	.	.	.	.	.	.	.	.	.	.	.	.	.	.	.	.	.	.	.	.	.	.	.	.	.	.	.	.	.
IE	BR-ES	MF423374/2017/PA196/Domingos Martins/V	.	.	.	.	.	.	.	.	.	.	.	.	.	.	.	.	.	.	.	.	.	.	.	.	.	.	.	.	.	.	.	.	.	.	.	.	.	.	.	.	.	.	.	.	.	.	.	.	.	.	.	.	.	.	.	.	.	.	.	.	.	.	.	.	.	.	.	.	.
IE	BR-ES	MF538782/2017/H190/São Fidelis/H	.	.	.	.	.	.	.	.	.	.	.	.	.	.	.	.	.	.	.	.	.	.	.	.	.	.	.	.	.	.	.	.	.	.	.	.	.	.	.	.	.	.	.	.	.	.	.	.	.	.	.	.	.	.	.	.	.	.	.	.	.	.	.	.	.	.	.	.	.
IE	BR-ES	MF538784/2017/H196/Porciuncula/H	.	.	.	.	.	.	.	.	.	.	.	.	.	.	.	.	.	.	.	.	.	.	.	.	.	.	.	.	.	.	.	.	.	.	.	.	.	.	.	.	.	.	.	.	.	.	.	.	.	.	.	.	.	.	.	.	.	.	.	.	.	.	.	.	.	.	.	.	.
IE	BR-ES	MF170977/B017/Conceicao do Castelo/H	.	.	.	.	.	.	.	.	.	.	.	.	.	.	.	.	.	.	.	.	.	.	.	.	.	.	.	.	.	.	.	.	.	.	.	.	.	.	.	.	.	.	.	.	.	.	.	.	.	.	.	.	.	.	.	.	.	.	.	.	.	.	.	.	.	.	.	.	.
IE	BR-ES	MF170968/2017/Marechal Floriano/H	.	.	.	.	.	.	.	.	.	.	.	.	.	.	.	.	.	.	.	.	.	.	.	.	.	.	.	.	.	.	.	.	.	.	.	.	.	D	.	F	.	.	.	.	.	.	.	.	.	.	.	.	.	.	.	.	.	.	.	.	.	.	.	.	.	.	G	.	.
IE	BR-ES	MF170976/2017/Laranja daTerra/H	.	.	.	.	.	.	.	.	.	.	.	.	.	.	.	.	.	.	.	.	.	.	.	.	.	.	.	.	.	.	.	.	.	.	.	.	.	.	.	.	.	.	.	.	.	.	.	.	.	.	.	.	.	.	.	.	.	.	.	.	.	.	.	.	.	.	.	.	.
IE	BR-ES	KY885001/2017/505/DomingosMartinsil/M	.	.	.	.	.	.	.	.	.	.	.	.	.	.	.	.	.	.	.	.	.	.	.	.	.	.	.	.	.	.	.	.	.	.	.	.	.	.	.	.	.	.	.	.	.	.	.	.	.	.	.	.	.	.	.	.	.	.	.	.	.	.	.	.	.	.	.	.	.
IE	BR-ES	MF170978/2017/Santa Maria de Jetiba/H	.	.	.	.	.	.	.	.	.	.	.	.	.	.	.	.	.	.	.	.	.	.	.	.	.	.	.	.	.	.	.	.	.	.	.	.	.	.	.	.	.	.	.	.	.	.	.	.	.	.	.	.	.	.	.	.	.	.	.	.	.	.	.	.	.	.	.	.	.
IE	BR-ES	MF170979/2017/Santa Maria de Jetiba /H	.	.	.	.	.	.	.	.	.	.	.	.	.	.	.	.	.	.	.	.	.	.	.	.	.	.	.	.	.	.	.	.	.	.	.	.	.	.	.	.	.	.	.	.	.	.	.	.	.	.	.	.	.	.	.	.	.	.	.	.	.	.	.	.	A	.	.	.	.
IE	BR-ES	MF170981/2017/Santa Leopoldina/H	.	.	.	.	.	.	.	.	.	.	.	.	.	.	.	.	.	.	.	.	.	.	.	.	.	.	.	.	.	.	.	.	.	.	.	.	.	.	.	.	.	.	.	.	.	F	.	.	.	.	.	.	.	.	.	.	.	.	.	.	.	.	.	.	.	.	.	.	.
IE	BR-ES	MF170970/2017/Domingos Martins/H	.	.	.	.	.	.	.	.	.	.	.	.	.	.	.	.	.	.	.	.	.	.	.	.	.	.	.	.	.	.	.	.	.	.	.	.	.	.	.	.	.	.	.	.	.	F	.	.	.	.	.	.	.	.	.	.	.	.	.	.	.	.	.	.	.	.	.	.	.
IE	BR-ES	MK333801/2017/Santa Leopoldina/V	.	.	.	.	.	.	.	.	.	.	.	.	.	.	.	.	.	.	.	.	.	.	.	.	.	.	.	.	.	.	.	.	.	.	.	.	.	.	.	.	.	.	.	.	.	.	.	.	.	.	.	.	.	.	.	.	.	.	.	.	.	.	.	.	.	.	.	.	.
IE	BR-ES	MF170972/2017/Cariacica /H	.	.	.	.	.	.	.	.	.	.	.	.	.	.	.	.	.	.	.	.	.	.	.	.	.	.	.	.	.	.	.	.	.	.	.	.	.	.	.	.	.	.	.	.	.	.	.	.	.	.	.	.	.	.	.	.	.	.	.	.	.	.	.	.	.	.	.	.	.
IE	BR-ES	MF170973/2017/Domingos Martins /H	.	.	.	.	.	.	.	.	.	.	.	.	.	.	.	.	.	.	.	.	.	.	.	.	.	.	.	.	.	.	.	.	.	.	.	.	.	.	.	.	.	.	.	.	.	.	.	.	.	.	.	.	.	.	.	.	.	.	.	.	.	.	.	.	.	.	.	.	.
IE	BR-ES	MK333802/ES2750/2017/Santa Leopoldina/V	.	.	.	.	.	.	.	.	.	.	.	.	.	.	.	.	.	.	.	.	.	.	.	I	.	.	.	.	.	.	.	.	.	.	.	.	.	.	.	.	.	.	.	.	.	.	.	.	.	.	.	.	.	.	.	.	.	.	.	.	.	.	.	.	.	.	.	.	.
IE	BR-ES	MF170974/2017/Cariacica /H	.	.	.	.	.	.	.	.	.	.	.	.	.	.	.	.	.	.	.	.	.	.	.	I	.	.	.	.	.	.	.	.	D	.	.	.	.	.	.	.	.	.	.	.	.	.	.	.	.	.	.	.	.	.	.	.	.	.	.	.	.	.	.	.	.	.	.	.	.
IE	BR-RJ	MF423376/2017/RJ94/Macae-Sana/M	.	.	.	.	.	.	.	.	.	.	.	.	.	.	.	.	.	.	.	.	.	.	.	.	.	.	.	.	.	.	.	.	.	.	.	.	.	.	.	.	.	.	.	.	.	.	.	.	.	.	.	.	.	.	.	.	.	.	.	.	.	.	.	.	.	.	.	.	.
IE	BR-RJ	MF538783/2017/H191/Casimiro de Abreu/H	.	.	.	.	.	.	.	.	.	.	.	.	.	.	.	.	.	.	.	.	.	.	.	.	.	.	.	.	.	.	.	.	.	.	.	.	.	.	.	.	.	.	.	.	.	.	.	.	.	.	.	.	.	.	.	.	.	.	.	.	.	.	.	.	.	.	.	.	.
IE	BR-RJ	MF434851/2017/H199/Silva Jardim/H	.	.	.	.	.	.	.	.	.	.	.	.	.	.	.	.	.	.	S	.	.	.	.	.	.	.	.	.	.	.	.	.	.	.	.	.	.	.	.	.	.	.	.	.	.	.	.	.	.	.	.	.	.	.	.	.	.	.	.	.	.	.	.	.	.	.	.	.	.
IE	BR-RJ	MK333805/IG3036/2018/Ilha Grande/V	.	.	.	.	.	.	.	.	.	.	.	.	.	.	.	.	.	.	.	.	.	.	.	.	.	.	.	.	.	.	.	.	.	.	.	.	.	.	.	.	.	.	.	.	.	.	.	.	.	.	.	.	.	.	.	.	.	.	.	.	.	.	.	.	.	.	.	.	.
IE	BR-RJ	MF538786/2017/RJ104/Guapimirim/M	.	.	.	.	.	.	.	.	.	.	.	.	.	.	.	.	.	.	.	.	.	.	.	.	.	.	.	.	.	.	.	.	.	.	.	.	.	.	.	.	.	.	.	.	.	.	.	.	.	.	.	.	.	.	.	.	.	.	.	.	.	.	.	.	.	.	.	.	.
IE	BR-RJ	MF423375/2017/RJ87/MacaeAtalaia/M	.	.	.	.	.	.	.	.	.	.	.	.	.	.	.	.	.	.	.	.	.	.	.	.	.	.	.	.	.	.	.	.	.	.	.	.	.	.	.	.	.	.	.	.	.	.	.	.	.	.	.	.	.	.	.	.	.	.	.	.	.	.	.	.	.	.	.	.	.
IE	BR-ES	MF170980/2017/Alfredo Chaves/H	.	.	.	.	.	.	.	.	.	.	.	.	.	.	.	.	.	.	.	.	.	.	.	.	.	.	.	.	.	.	.	.	.	.	.	.	.	.	.	.	.	.	.	.	.	.	.	.	.	.	.	.	.	.	.	.	.	.	.	.	.	.	S	D	A	.	.	.	.
IE	BR-MG-E	MH018096/2017/JoseRaydan/M	.	.	.	.	.	.	.	.	.	.	.	.	.	.	.	.	.	.	.	.	.	.	.	.	.	.	.	.	.	.	.	.	.	.	.	.	.	.	.	.	.	.	.	.	.	.	.	.	.	.	.	.	.	.	.	.	.	.	.	.	.	.	.	.	.	.	.	.	.
IE	BR-MG-E	MH018079/2017/Itambacuri/H	.	.	.	.	.	.	.	.	.	.	.	.	.	.	.	.	.	.	.	.	.	.	.	.	.	.	.	.	.	.	.	.	.	.	.	.	.	.	.	.	.	.	.	.	.	.	.	.	.	.	.	.	.	.	.	.	.	.	.	.	.	.	.	.	.	.	.	.	.
IE	BR-MG-E	MH018095/2017/Sabinopolis/M	.	.	.	.	.	.	.	.	.	.	.	.	.	.	.	.	.	.	.	.	.	.	.	.	.	.	.	.	.	.	.	.	.	.	.	.	.	.	.	.	.	.	.	.	.	.	.	.	.	.	.	.	.	.	.	.	.	.	.	.	.	.	.	.	.	.	.	.	.
IE	BR-MG-E	MH018076/2017/Setubinha/H	.	.	.	.	.	.	.	.	.	.	.	.	.	.	.	.	.	.	.	.	.	.	.	.	.	.	.	.	.	.	.	.	.	.	.	.	.	.	.	.	.	.	.	.	.	.	.	.	.	.	.	.	.	.	.	.	.	.	.	.	.	.	.	.	.	.	.	.	.
IE	BR-MG-E	MH018083/2017/AbreCampo/M	.	.	.	.	.	.	.	.	.	.	.	.	.	.	.	.	.	.	.	.	.	.	.	.	.	.	.	.	.	.	.	.	.	.	.	.	.	.	.	.	.	.	.	.	.	.	.	.	.	.	.	.	.	.	.	.	.	.	.	.	.	.	.	.	.	.	.	.	.
IE	BR-MG-E	MH018078/2017/Novo Cruzeiro/H	.	.	.	.	.	.	.	.	.	.	.	.	.	.	.	.	.	.	.	.	.	.	.	.	.	.	.	.	.	.	.	.	D	.	.	.	.	.	.	.	.	.	.	.	.	.	.	.	.	.	.	.	.	.	.	.	.	.	.	.	.	.	.	.	.	.	.	.	.
IE	BR-MG-E	MH018091/2017/Novo Cruzeiro/H	.	.	.	.	.	.	.	.	.	.	.	.	.	.	.	.	.	.	.	.	.	.	.	.	.	.	.	.	.	.	.	.	.	.	.	.	.	.	.	.	.	.	.	.	.	.	.	.	.	.	.	.	.	.	.	.	.	.	.	.	.	.	.	.	.	.	.	.	.
IE	BR-MG-E	MH018088/2017/Teofilo Otoni/H	.	.	.	.	.	.	.	.	.	.	.	.	.	.	.	.	.	.	.	.	.	.	.	.	.	.	.	.	.	.	.	.	.	.	.	.	.	.	.	.	.	.	.	.	.	.	.	.	.	.	.	.	.	.	.	.	.	.	.	.	.	P	.	.	.	.	.	.	.
IE	BR-MG-E	MH018093/2017/StaBarbaraLeste/H	.	.	.	.	.	.	.	.	.	.	.	.	.	.	.	.	.	.	.	.	.	.	.	.	.	.	.	.	.	.	.	.	.	.	.	.	.	.	.	.	.	.	.	.	.	.	.	.	.	E	.	.	.	.	.	.	.	.	.	.	.	.	.	.	.	.	.	.	.
IE	BR-MG-E	MH018084/2017/Ladainha/M	.	.	.	.	.	.	.	.	.	.	.	.	.	.	.	.	.	.	.	.	.	.	.	.	.	.	.	.	.	.	.	.	.	.	.	.	.	.	.	.	.	.	.	.	.	.	.	.	.	.	.	.	.	.	.	.	.	.	.	.	.	.	.	.	.	.	.	.	.
IE	BR-MG-E	MH018089/2017/Ladainha/H	.	.	.	.	.	.	.	.	.	.	.	.	.	.	.	.	.	.	.	.	.	.	.	.	.	.	.	.	.	.	.	.	.	.	.	.	.	.	.	.	.	.	.	.	.	.	.	.	.	.	.	.	.	.	.	.	.	.	.	.	.	.	.	.	.	.	.	.	.
IE	BR-MG-E	MH018092/2017/Itambacuri/H	.	.	.	.	.	.	.	.	.	.	.	.	.	.	.	.	.	.	.	.	.	.	.	.	.	.	.	.	.	.	.	.	.	.	.	.	.	.	.	.	.	.	.	.	.	.	.	.	.	.	.	.	.	.	.	.	.	.	.	.	.	.	.	.	.	.	.	.	.
IE	BR-MG-E	MH018080/2017/Ladainha/H	.	.	.	.	.	.	.	.	.	.	.	.	.	.	.	.	.	.	.	.	.	.	.	.	.	.	.	.	.	.	.	.	.	.	.	.	.	.	.	.	.	.	.	.	.	.	.	.	.	.	.	.	.	.	.	.	.	.	.	.	.	.	.	.	.	.	.	.	.
IE	BR-MG-E	MH018090/2017/Caratinga/H	.	.	.	.	.	.	.	.	.	.	.	.	.	.	.	.	.	.	.	.	.	.	.	.	.	.	.	.	.	.	.	.	.	.	.	.	.	.	.	.	.	.	.	.	.	.	.	.	.	.	.	.	.	.	.	.	.	.	.	.	.	.	.	.	.	.	.	.	.
IE	BR-MG-E	MH484434/2017/SaoJoaoEvangelista/H	.	.	.	.	.	.	.	.	.	.	.	.	.	.	.	.	.	.	.	.	.	.	.	.	.	.	.	.	.	.	.	.	.	.	.	.	.	.	.	.	.	.	.	.	.	.	.	.	.	.	S	.	.	.	.	.	.	.	.	.	.	.	.	.	.	.	.	.	.
IE	BR-MG-E	MH484427/2017/FeliciodosSantos/M	.	.	.	.	.	.	.	.	.	.	.	.	.	.	.	.	.	.	.	.	.	.	.	.	.	.	.	.	.	.	.	.	.	.	.	.	.	.	.	.	.	.	.	.	.	.	.	.	.	.	.	.	.	.	.	.	.	.	.	.	.	.	.	.	.	.	.	.	.
IE	BR-MG-N	MF465805/2017/Januaria/H	.	.	.	.	.	.	.	.	.	.	.	.	.	.	.	.	.	.	.	.	.	.	.	.	.	.	.	.	.	.	.	.	.	.	.	.	.	.	.	.	.	.	.	.	.	.	.	.	.	.	.	.	.	.	.	.	.	.	.	.	.	.	.	.	.	.	.	.	R
IE	BR-MG-N	MH484429/2017/ChapadaGaucha/M	.	.	.	.	.	.	.	.	.	.	.	.	.	.	.	.	.	.	.	.	.	.	.	.	.	.	.	.	.	.	.	.	.	.	.	.	.	.	.	.	.	.	.	.	.	.	.	.	.	.	.	.	.	.	.	.	.	.	.	.	.	.	.	.	.	.	.	.	R
IE	BR-BA	MH018099/2017/Caordeiros/M	.	.	.	.	.	.	.	.	.	.	.	.	.	.	.	I	.	.	.	.	.	.	.	.	.	.	.	.	.	.	.	.	.	.	.	.	.	.	.	.	.	.	.	.	.	.	.	.	.	.	.	.	.	.	.	.	.	.	.	.	.	.	.	.	.	.	.	.	R
IE	BR-SP	MK333798/2018/isolate/São Paulo/H	.	.	.	.	.	.	.	.	.	.	.	.	.	.	.	.	.	.	.	.	H	.	.	.	.	.	.	.	.	.	.	.	.	.	A	.	.	.	.	.	.	.	.	.	.	.	.	.	.	.	.	.	T	.	.	.	.	.	.	.	.	.	.	.	.	.	.	.	.
IE	BR-SP	MK333799/2018/isolate/São Paulo/H	.	.	.	.	.	.	.	.	.	.	.	.	.	.	.	.	.	.	.	.	.	.	.	.	.	.	.	.	.	.	.	.	.	.	.	.	.	.	.	.	.	.	.	.	.	.	.	.	.	.	.	.	T	.	.	.	.	.	.	.	.	.	.	.	.	.	.	.	.
IE	BR-MG-SW	MH018064/2017/StaRitadeCaldas/M	.	.	.	.	.	.	.	.	.	.	.	.	.	.	.	.	.	.	.	.	.	.	.	.	.	.	.	.	.	.	.	.	.	.	.	.	.	.	.	.	.	.	.	.	.	.	.	.	.	.	.	.	T	.	.	.	.	.	.	.	.	.	.	.	.	.	.	.	.
IE	BR-MG-SW	MH018067/2017/Caldas/M	.	.	.	.	.	.	.	.	.	.	.	.	.	.	.	.	.	.	.	.	.	.	.	.	.	.	.	.	.	.	.	.	.	.	.	.	.	.	.	.	.	A	.	.	.	.	.	.	.	.	.	.	T	.	.	.	.	.	.	.	.	.	.	.	.	.	.	.	.
IE	BR-MG-SW	MH018066/2017/OuroFino/M	.	.	.	.	.	.	.	.	.	.	.	.	.	.	.	.	.	.	.	.	.	.	.	.	.	.	.	.	.	.	.	.	.	.	.	.	.	.	.	.	.	.	.	.	.	.	.	.	.	.	.	.	T	.	.	.	.	.	.	.	.	.	.	.	.	.	.	.	.
IE	BR-MG-SW	MH018065/2017/Delfinopolis/M	.	.	.	.	.	.	.	.	.	.	.	.	.	.	.	.	.	.	.	.	.	.	.	.	.	.	.	.	.	.	.	.	.	.	.	.	.	.	.	.	.	.	.	.	.	.	.	.	.	.	.	.	.	.	.	.	.	.	.	.	.	.	.	.	.	.	.	.	.
IE	BR-MG-SW	MH018082/2017/Claraval/M	.	.	.	.	.	.	.	.	.	.	.	.	.	.	.	.	.	.	.	.	.	.	.	.	.	.	.	.	.	.	.	.	.	.	.	.	.	.	.	.	.	.	.	.	.	.	.	.	.	.	.	.	.	.	.	.	.	.	.	.	.	.	.	.	.	.	.	.	.
IE	BR-MG-SW	MF170971/2017/SaoRoquedeMinas/M	.	.	.	.	.	.	.	.	.	.	.	.	.	.	.	.	.	.	.	.	.	.	.	.	.	.	.	.	.	.	.	.	.	.	.	.	.	.	.	.	.	.	.	.	.	.	.	.	.	.	.	.	.	.	.	.	.	.	.	.	.	.	.	.	.	.	.	.	.
IE	BR-MG-SW	MH484430/2017/Sacramento/M	.	.	.	.	.	.	.	.	.	.	.	.	.	.	.	.	.	.	.	.	.	.	.	.	.	.	.	.	.	.	.	.	.	.	.	.	.	.	.	.	.	.	.	.	.	.	.	.	.	.	.	.	.	.	.	.	.	.	.	.	.	.	.	.	.	.	.	.	.
IE	BR-GO	MK728873/2017/GO21/Amorinópolis /H	.	.	.	.	.	.	.	.	.	.	.	.	.	.	.	.	.	.	.	.	.	.	.	.	.	.	.	.	.	.	.	.	.	.	.	.	.	.	.	.	.	.	.	.	.	.	.	.	.	.	.	.	.	.	.	.	.	.	.	.	.	.	.	.	.	.	.	.	.
IE	BR-GO	MK333804/2015/GO27/Novo Brasil/M	.	.	.	.	.	.	.	.	.	.	.	.	.	.	.	.	.	.	.	.	.	.	.	.	.	.	.	.	.	.	.	.	.	.	.	.	.	.	.	.	.	.	.	.	.	.	.	.	.	.	.	.	.	.	.	.	.	.	.	.	.	.	.	.	.	.	.	.	.
IE	BR-RS	KY861728/2008/Caibate/M	.	.	.	.	V	.	.	.	.	.	.	.	.	.	.	.	.	.	.	.	.	.	N	.	S	.	.	.	.	.	.	.	.	.	.	.	.	.	.	.	.	.	.	.	.	.	.	.	.	E	.	R	.	R	.	.	.	.	.	.	.	.	.	.	.	.	.	.	.
IE	BR-GO	MK333803/GO05/2017/Nova Crixás/M	.	.	.	.	V	.	.	.	.	.	.	.	.	.	.	.	.	.	.	.	.	.	.	.	.	.	.	.	.	.	.	.	.	H	.	.	.	.	.	.	.	.	.	.	.	.	.	.	.	E	.	R	.	.	.	.	.	.	.	.	.	.	.	.	.	.	.	.	.
IE	BR-RR	JF912190/BeH655417/2002/Alto Alegre/H	.	.	.	.	V	.	.	.	.	.	.	.	.	.	.	.	.	.	.	.	.	.	.	.	.	.	V	.	.	.	V	.	.	.	.	.	.	.	.	.	.	.	.	.	.	.	.	.	.	E	.	R	.	.	.	.	.	N	.	.	.	.	.	.	.	.	.	.	.
IE	Ven	KM388817/Venezuela/2A/2004/M	.	.	.	.	V	A	.	.	.	T	.	.	.	.	.	.	.	.	.	.	.	.	.	A	.	.	.	.	.	.	.	.	.	.	.	.	.	.	.	.	.	.	.	.	.	.	.	.	.	E	.	R	.	.	.	.	.	.	.	.	.	.	.	.	.	.	.	.	.
IE	Ven	KM388814/Venezuela/6A/2005/H	.	.	.	.	V	.	.	.	.	.	.	.	.	.	.	.	.	.	.	.	.	.	.	.	.	.	.	.	.	I	.	.	.	.	.	.	.	.	.	.	T	.	.	.	.	.	.	T	.	E	.	R	.	.	.	.	.	.	.	.	.	.	.	.	.	.	.	.	.
IE	Ven	KM388818/Venezuela/8A/2006/M	.	.	.	.	V	.	.	.	.	.	.	.	.	.	.	.	.	.	.	.	.	.	.	.	.	.	.	.	.	I	.	.	.	.	.	.	.	.	.	.	T	.	.	.	.	.	.	T	.	E	.	R	.	.	.	.	.	.	.	K	.	.	.	.	.	.	.	.	.
IE	Ven	KM388815/Venezuela/9A/2007/M	.	.	.	.	V	.	.	.	.	.	.	.	.	.	.	.	.	.	.	.	.	.	.	.	.	.	.	.	.	I	.	I	S	.	.	.	.	.	.	.	T	.	.	.	.	.	.	T	.	E	.	R	.	.	.	V	.	.	.	.	.	.	.	.	.	.	.	.	.
IE	Ven	KM388816/Venezuela/10A/2010/M	.	.	.	.	V	.	.	.	.	.	.	.	.	.	.	.	.	.	.	.	.	.	.	.	.	.	.	.	.	.	.	.	.	.	.	.	.	.	.	.	.	.	.	.	.	.	.	.	.	E	.	R	.	.	.	.	.	.	.	.	.	.	.	.	.	.	.	.	.
TT	TT	HM582851/Trinidad and Tobago/TVP11767/2009/M	.	A	K	.	V	.	.	.	.	.	.	.	.	.	.	.	.	.	.	.	.	R	.	.	.	.	.	.	.	.	.	.	.	.	.	.	.	.	.	.	.	.	.	.	.	.	.	.	R	E	.	R	.	.	.	.	.	.	S	.	.	.	.	.	.	.	.	.	.
1D	BR-MG	MG969501/2001/Bom Despacho/H	.	A	K	.	V	.	.	.	.	.	.	.	.	.	.	.	.	.	.	.	.	.	.	.	S	.	.	V	.	.	.	.	.	.	.	.	.	.	.	.	.	.	.	.	.	.	.	.	.	E	.	R	.	.	.	.	.	.	.	.	.	.	.	.	.	.	.	.	.
1D	BR-RS	JF912189/2001/S Miguel Missões/V	.	A	K	.	V	.	.	.	.	.	.	V	.	.	.	.	.	.	.	.	.	.	.	.	S	.	.	V	.	.	.	.	.	.	.	.	.	.	.	.	.	.	.	.	.	.	.	.	.	E	.	R	.	.	S	.	.	.	.	.	.	.	.	.	.	.	.	.	.
1D	BR-GO	JF912188/2000/AltoParaiso/H	.	A	K	.	V	.	.	.	.	.	.	.	.	.	.	.	.	.	.	.	.	.	.	.	S	.	.	V	.	.	.	.	.	.	.	.	.	.	.	.	.	.	.	.	.	.	.	.	.	E	.	R	.	.	.	.	.	.	.	.	.	.	.	.	.	.	.	.	.
1D	BR-GO	JF912187/2000/Goias/H	.	A	K	.	V	.	.	.	S	.	.	.	.	.	.	.	.	.	.	.	.	.	.	.	S	.	.	V	.	.	.	.	.	.	.	.	.	.	.	.	.	.	.	.	.	.	.	.	.	E	.	R	.	.	.	.	T	.	.	.	.	.	.	.	.	.	.	.	.
1C	BR-MG	JF912186/1994/ Arinos/H	R	A	K	.	V	.	.	S	.	.	.	.	K	.	.	.	.	S	.	.	.	.	.	.	.	.	.	.	T	.	.	.	S	.	.	.	.	V	I	.	.	.	K	.	.	.	L	.	.	E	.	R	.	.	.	.	.	.	.	.	.	.	.	.	.	A	.	.	.
1C	BR-PA	JF912184/1987/ Breves/H	.	A	K	A	V	.	.	.	.	.	.	.	K	R	.	.	.	S	.	S	.	.	.	.	.	.	.	.	.	.	.	.	.	.	.	.	.	.	I	.	.	.	K	.	T	.	L	.	.	E	.	R	.	.	.	.	.	.	.	.	.	.	.	.	.	.	.	.	.
1C	BR-PA	JF912183/1984/ S.Domingos do Capim/H	.	A	K	.	V	.	.	.	.	.	.	.	K	.	.	.	.	S	.	.	.	.	.	.	.	.	.	.	.	.	.	.	.	.	.	.	.	.	I	.	.	.	K	.	.	.	L	.	.	E	.	R	.	.	.	.	.	.	.	.	.	.	.	.	.	.	.	.	.
1C	BR-GO	JF912179/ 1980/Uruaçu/V	.	A	K	A	V	.	.	.	.	.	.	.	K	.	.	.	S	S	.	.	.	.	.	.	.	.	.	.	.	.	.	.	.	.	.	.	.	.	I	.	.	.	K	.	.	.	L	.	.	E	.	R	.	.	.	.	.	.	.	.	.	.	.	.	.	.	.	.	.
1B	BR-MS	JF912185/BR-MS/ BeAR513008/1992/Sidrolândia/V	.	A	K	.	V	.	K	.	.	.	.	.	.	.	.	.	.	.	.	.	.	.	.	.	.	R	.	V	.	.	.	.	.	.	.	T	A	.	.	.	.	.	.	.	.	.	.	.	.	E	.	R	.	.	.	.	.	.	.	.	K	.	.	.	.	.	.	M	.
1B	BR-PA	JF912182/1984/Monte Alegre/H	.	A	K	.	V	.	K	.	.	.	A	.	.	.	.	.	.	.	.	.	.	.	.	.	.	.	.	.	.	.	.	.	.	.	.	T	.	.	.	.	.	.	.	.	.	.	.	.	R	E	.	R	.	.	.	.	.	.	.	.	.	.	.	.	.	.	.	.	.

Supplementary Figure 3 cont.

		Viral proteins	NS4A		NS4B										NS5																																
Subclade		YFV sample / polyprotein amino acid position	2137	2176	2276	2288	2311	2334	2372	2408	2426	2433	2502	2503	2584	2601	2607	2614	2630	2638	2640	2644	2647	2648	2679	2736	2803	2903	2907	3031	3149	3152	3158	3209	3215	3234	3245	3273	3310	3329	3341	3345	3346	3363	3372	3375	3384
1E	BR-ES	KY885000/ES504/2017/DomingosMartins/M	L	I	I	F	H	S	A	N	K	L	K	T	T	R	R	E	I	V	R	I	D	T	S	T	S	K	I	A	A	K	A	G	S	I	V	S	K	T	K	D	I	M	H	L	I
	BR-RJ	MK333809/VL2926/2018/Valença/V	.	V	.	.	.	.	.	.	.	.	.	.	.	.	.	.	.	.	.	.	.	.	.	.	.	.	.	.	.	.	.	.	.	.	.	.	.	.	.	.	.	.	.	.	.
	BR-MG-SE	MK333808/MG3155/2018/Belmiro Braga/V	.	V	.	.	.	.	.	.	.	.	.	.	.	.	.	.	.	.	.	.	.	.	.	.	.	.	.	.	.	.	.	.	.	.	.	.	.	.	.	.	.	.	.	.	.
	BR-MG-SE	MK333807/MG3121/2018/Belmiro Braga/V	.	V	.	.	.	.	.	.	.	.	.	.	.	.	.	.	.	.	.	.	.	.	.	.	.	.	.	.	.	.	.	.	.	.	.	.	.	.	.	.	.	.	.	.	.
	BR-MG-SE	MK333806/JF2793/2018/Juiz de Fora/V	.	V	.	.	.	.	.	.	.	.	.	.	.	.	.	.	.	.	.	.	.	.	.	.	.	.	.	.	.	.	.	.	.	.	.	.	.	.	.	.	.	.	.	.	.
	BR-RJ	MF423377/2017/RJ95/Carmo/M	.	V	.	.	.	.	.	.	.	.	.	.	.	.	.	.	.	.	.	.	.	.	.	.	.	.	.	.	.	.	.	.	.	.	.	.	.	.	.	.	.	.	.	.	.
	BR-RJ	MF423378/2017/RJ96/Carmo/M	.	V	.	.	.	.	.	.	.	.	.	.	.	.	.	.	.	.	.	.	.	.	.	.	.	.	.	.	.	.	.	.	.	.	.	.	.	.	.	.	.	.	.	.	.
	BR-RJ	MF538785/2017/RJ97/Petropolis/M	.	V	.	.	.	.	.	.	.	.	.	.	.	.	.	.	.	.	.	.	.	.	.	.	.	.	.	.	.	.	.	.	.	.	.	.	.	.	.	.	.	.	.	.	.
	BR-ES	MF423373/2017/PA193/Domingos Martins/V	.	V	.	.	.	.	.	.	.	.	.	.	.	.	.	.	.	.	.	.	.	.	.	.	.	.	.	.	.	.	.	.	.	.	.	.	.	.	.	.	.	.	.	.	.
	BR-ES	MF170975/2017/Brejetuba /H	.	V	.	.	.	.	.	.	.	.	.	.	.	.	.	.	.	.	.	.	.	.	.	.	.	.	.	.	.	.	.	.	.	.	.	.	.	.	.	.	.	.	.	.	.
	BR-ES	MK333800/ ES03/2017/Domingos Martins/M	.	.	.	.	.	.	.	.	.	.	.	.	.	.	.	.	.	.	.	.	.	.	.	.	.	.	.	.	.	.	.	.	.	.	.	.	.	.	.	.	.	.	.	.	.
	BR-ES	MF423374/2017/PA196/Domingos Martins/V	.	.	.	.	.	.	.	.	.	.	.	.	.	.	.	.	.	.	.	.	.	.	.	.	.	.	.	.	.	.	.	.	.	.	.	.	.	.	.	.	.	.	.	.	.
	BR-ES	MF538782/2017/H190/São Fidelis/H	.	.	.	.	.	.	.	.	.	.	.	.	.	.	.	.	.	.	.	.	.	.	.	.	.	.	.	.	.	.	.	.	.	.	.	.	.	.	.	.	.	.	.	.	.
	BR-ES	MF538784/2017/H196/Porciuncula/H	.	.	.	.	.	.	.	.	.	.	.	.	.	.	.	.	.	.	.	.	.	.	.	.	.	.	.	.	.	.	.	.	.	.	.	.	.	.	.	.	.	.	.	.	.
	BR-ES	MF170977/B017/Conceicao do Castelo/H	.	.	.	.	.	.	.	.	.	.	.	.	.	.	.	.	.	.	.	.	.	.	.	.	.	.	.	.	.	.	.	.	.	.	.	.	.	.	.	.	.	.	.	.	.
	BR-ES	MF170968/2017/Marechal Floriano/H	.	.	.	.	.	.	.	.	.	.	.	.	.	.	.	.	.	.	.	.	.	.	.	.	.	.	.	.	.	.	.	E	.	.	.	.	.	.	.	.	.	.	.	.	.
	BR-ES	MF170976/2017/Laranja daTerra/H	.	.	.	.	.	.	.	.	.	.	.	.	.	.	.	.	.	.	.	.	.	.	.	.	.	.	.	.	.	.	.	.	.	.	.	.	.	.	.	.	.	.	.	.	.
	BR-ES	KY885001/2017/505/DomingosMartinsil/M	.	.	.	.	.	.	.	.	.	.	.	.	.	.	.	.	.	.	.	.	.	.	.	.	.	.	.	.	.	.	.	.	.	.	.	.	.	.	.	.	.	.	.	.	.
	BR-ES	MF170978/2017/Santa Maria de Jetiba/H	.	.	.	.	.	.	.	.	.	.	.	.	.	.	.	.	.	.	.	.	.	.	.	.	.	.	.	.	.	.	.	.	.	.	.	.	.	.	.	.	.	.	.	.	.
	BR-ES	MF170979/2017/Santa Maria de Jetiba /H	.	.	.	.	.	.	.	.	.	.	.	.	.	.	.	.	.	.	.	.	.	.	.	.	.	.	.	.	.	.	.	.	.	.	.	.	.	.	.	.	.	.	.	.	M
	BR-ES	MF170981/2017/Santa Leopoldina/H	.	.	.	.	.	.	.	.	.	.	.	.	.	.	.	.	.	.	.	.	.	.	.	.	.	.	.	.	.	.	.	.	.	.	.	.	.	.	.	.	.	.	.	.	.
	BR-ES	MF170970/2017/Domingos Martins/H	.	.	.	.	.	.	.	.	.	.	.	.	.	.	.	.	.	.	.	.	.	.	.	.	.	.	.	.	.	.	.	.	.	.	.	.	.	.	.	.	.	.	.	.	.
	BR-ES	MK333801/2017/Santa Leopoldina/V	.	.	.	.	.	.	.	.	.	.	.	.	.	.	.	.	.	.	.	.	.	.	.	.	.	.	.	.	.	.	.	.	.	.	.	.	.	.	.	.	.	.	.	.	.
	BR-ES	MF170972/2017/Cariacica /H	.	.	.	.	.	.	.	.	.	.	.	.	.	.	.	.	.	.	.	.	.	.	.	.	.	.	.	.	.	.	.	.	.	.	.	.	.	.	.	.	.	.	.	.	.
	BR-ES	MF170973/2017/Domingos Martins /H	.	.	.	.	.	.	.	.	.	.	.	.	.	.	.	.	.	.	.	.	.	.	.	.	.	.	.	.	.	.	.	.	.	.	.	.	.	.	.	.	.	.	.	.	.
	BR-ES	MK333802/ES2750/2017/Santa Leopoldina/V	.	.	.	.	.	.	.	.	.	.	.	.	.	.	.	.	.	.	.	.	.	.	.	.	.	.	.	.	.	.	.	.	.	.	.	.	.	.	.	.	.	.	.	.	.
	BR-ES	MF170974/2017/Cariacica /H	.	.	.	.	.	.	.	.	.	.	.	.	.	.	.	.	.	.	.	.	.	.	.	.	.	.	.	.	.	.	.	.	.	.	.	.	.	.	.	.	.	.	.	.	.
	BR-RJ	MF423376/2017/RJ94/Macae-Sana/M	.	.	.	.	.	.	.	.	.	.	.	.	.	.	.	.	.	.	.	.	.	.	.	.	.	.	.	.	.	.	.	.	.	.	.	.	.	.	.	.	.	.	.	.	.
	BR-RJ	MF538783/2017/H191/Casimiro de Abreu/H	.	V	.	.	.	.	.	.	.	.	.	.	.	.	.	.	.	.	.	.	.	.	.	.	.	.	.	.	.	.	.	.	.	.	.	.	.	.	.	.	.	.	.	.	.
	BR-RJ	MF434851/2017/H199/Silva Jardim/H	.	.	.	.	.	.	.	.	.	.	.	.	.	.	.	.	.	.	.	.	.	.	.	.	.	.	.	.	.	.	.	.	.	.	.	.	.	.	.	.	.	.	.	.	.
	BR-RJ	MK333805/IG3036/2018/Ilha Grande/V	.	.	.	.	.	.	.	.	.	.	.	.	.	.	.	.	.	.	.	.	.	.	.	.	.	.	.	.	.	.	.	.	.	.	.	.	.	.	.	.	.	.	.	.	.
	BR-RJ	MF538786/2017/RJ104/Guapimirim/M	.	.	.	.	.	.	.	.	.	.	.	.	.	.	.	.	.	.	.	.	.	.	.	.	.	.	.	.	.	.	.	.	.	.	.	.	.	.	.	.	.	.	.	.	.
	BR-RJ	MF423375/2017/RJ87/MacaeAtalaia/M	.	.	.	.	.	.	.	.	.	.	.	.	.	.	.	.	.	.	.	.	.	.	.	.	.	.	.	.	.	.	.	.	.	.	.	.	.	.	.	.	.	.	.	.	.
	BR-ES	MF170980/2017/Alfredo Chaves/H	.	.	.	.	.	A	.	.	.	.	.	.	.	.	.	.	.	.	.	.	.	.	.	.	.	.	.	.	.	.	.	.	.	.	.	.	.	.	.	.	.	.	.	.	.
	BR-MG-E	MH018096/2017/JoseRaydan/M	.	.	.	.	.	.	.	.	.	.	.	.	.	.	.	.	.	.	.	.	.	.	.	.	.	.	.	.	.	.	.	.	.	.	.	.	.	.	.	.	.	.	.	.	.
	BR-MG-E	MH018079/2017/Itambacuri/H	.	.	.	.	.	.	.	.	.	.	.	.	.	.	.	.	.	.	.	.	.	.	.	.	.	.	.	.	.	.	.	.	.	.	.	.	.	.	.	.	.	.	.	.	.
	BR-MG-E	MH018095/2017/Sabinopolis/M	.	.	.	.	.	.	.	.	.	.	.	.	.	.	.	.	.	.	.	.	.	.	.	.	.	.	.	.	.	.	.	.	.	.	.	.	.	.	.	.	.	.	.	.	.
	BR-MG-E	MH018076/2017/Setubinha/H	.	.	.	.	.	.	.	.	.	.	.	.	.	.	.	.	.	.	.	.	.	.	.	.	.	.	.	.	.	.	.	.	.	.	.	.	.	.	.	.	.	.	.	.	.
	BR-MG-E	MH018083/2017/AbreCampo/M	.	.	.	.	.	.	.	.	.	.	.	.	.	.	.	.	.	.	.	.	.	.	.	.	.	.	.	.	.	.	.	.	.	.	.	.	.	.	.	.	.	.	.	.	.
	BR-MG-E	MH018078/2017/Novo Cruzeiro/H	.	.	.	.	.	.	.	.	.	.	.	.	.	.	.	.	.	.	.	.	.	.	.	.	.	.	.	.	.	.	.	.	.	.	.	.	.	.	.	.	.	.	.	.	.
	BR-MG-E	MH018091/2017/Novo Cruzeiro/H	.	.	.	.	.	.	.	.	.	.	.	.	.	.	.	.	.	.	.	.	.	.	.	.	.	.	.	.	.	.	.	.	.	.	.	.	.	.	.	.	.	.	.	.	.
	BR-MG-E	MH018088/2017/Teofilo Otoni/H	.	.	.	.	.	.	.	.	.	.	.	.	.	.	Q	.	.	.	.	.	.	.	.	.	.	.	.	.	.	.	.	.	.	.	.	.	.	.	.	.	.	.	.	.	.
	BR-MG-E	MH018093/2017/StaBarbaraLeste/H	.	.	.	.	.	.	.	.	.	.	.	.	.	.	Q	.	.	.	.	.	.	.	.	.	.	.	.	.	.	.	.	.	.	.	.	.	.	.	.	.	.	.	.	.	.
	BR-MG-E	MH018084/2017/Ladainha/H	.	.	.	.	.	.	.	.	.	.	.	.	.	.	Q	.	.	.	.	.	.	.	.	.	.	.	.	.	.	.	.	.	.	.	.	.	.	.	.	.	.	.	.	.	.
	BR-MG-E	MH018089/2017/Ladainha/H	.	.	.	.	.	.	.	.	.	.	.	.	.	.	Q	.	.	.	.	.	.	.	.	.	.	.	.	.	.	.	.	.	.	.	.	.	.	.	.	.	.	.	.	.	.
	BR-MG-E	MH018092/2017/Itambacuri/H	.	.	.	.	.	.	.	.	.	.	.	.	.	.	.	.	.	.	.	.	.	.	.	.	.	.	.	.	.	.	.	.	.	.	.	.	.	.	.	.	.	.	.	.	.
	BR-MG-E	MH018080/2017/Ladainha/H	.	.	.	.	.	.	.	.	.	.	.	.	.	.	.	.	.	.	.	.	.	.	.	.	.	.	.	.	.	.	.	.	.	.	.	.	.	.	.	.	.	.	.	.	.
	BR-MG-E	MH018090/2017/Caratinga/H	.	.	.	.	.	.	.	.	.	.	.	.	.	.	.	.	.	.	.	.	.	.	.	.	.	.	.	.	.	.	.	.	.	.	.	.	.	.	.	.	.	.	.	.	.
	BR-MG-E	MH484434/2017/SaoJoaoEvangelista/H	.	.	.	.	.	.	.	.	.	.	.	.	.	.	.	.	.	.	.	.	.	.	.	.	.	.	.	.	.	.	.	.	.	.	.	.	.	.	.	.	.	.	.	.	.
	BR-MG-E	MH484427/2017/FeliciodosSantos/M	.	.	.	.	.	.	.	.	.	.	.	.	.	.	.	.	.	.	.	.	.	.	.	.	.	.	.	.	.	.	.	.	.	.	.	.	.	.	.	.	.	.	.	.	.
	BR-MG-N	MF465805/2017/Januaria/H	.	.	.	L	.	.	.	.	.	.	.	.	.	.	.	.	.	.	.	.	.	.	.	.	.	.	.	.	.	.	.	.	.	.	.	.	.	.	.	.	.	.	.	.	.
	BR-MG-N	MH484429/2017/ChapadaGaucha/M	.	.	.	.	.	.	.	.	.	.	.	.	.	.	.	.	.	.	.	.	.	.	.	.	.	.	.	.	.	.	.	.	.	.	.	.	.	.	.	.	.	.	.	.	.
	BR-BA	MH018099/2017/Caordeiros/M	.	.	.	.	.	.	.	.	.	.	.	.	.	.	.	.	.	.	.	.	.	.	.	.	.	.	.	.	.	.	.	.	.	.	.	.	.	.	.	.	.	.	.	.	.
	BR-SP	MK333798/2018/isolate/São Paulo/H	.	.	.	.	.	.	.	.	.	.	.	.	.	.	.	.	.	.	.	.	.	.	.	.	.	.	.	.	.	.	.	.	.	.	.	.	.	I	.	.	.	.	.	.	.
	BR-SP	MK333799/2018/isolate/São Paulo/H	.	.	.	.	.	.	.	.	.	.	.	.	.	.	.	.	.	.	.	.	.	.	.	.	.	.	.	.	.	.	.	.	.	.	.	.	.	I	.	.	.	.	.	.	.
	BR-MG-SW	MH018064/2017/StaRitadeCaldas/M	.	.	.	.	.	.	.	.	R	.	.	.	.	.	Q	.	.	.	.	.	.	.	.	.	.	.	.	.	.	.	.	.	.	.	.	.	.	.	.	.	.	.	.	.	.
	BR-MG-SW	MH018067/2017/Caldas/M	.	.	.	.	.	.	.	.	.	.	.	.	.	.	.	.	.	.	.	.	.	.	.	.	.	.	.	.	.	.	.	.	.	.	.	.	.	.	.	.	.	.	.	.	.
	BR-MG-SW	MH018066/2017/OuroFino/M	.	.	.	.	.	.	.	.	.	.	.	.	.	.	.	.	.	.	.	.	.	.	.	.	.	.	.	.	.	.	.	.	.	.	.	.	.	.	.	.	.	.	.	.	.
	BR-MG-SW	MH018065/2017/Delfinopolis/M	.	.	.	.	.	.	.	.	.	.	.	.	.	.	.	.	.	.	.	.	.	.	.	.	.	.	.	.	.	.	.	.	.	.	.	.	.	.	.	.	.	.	.	.	.
	BR-MG-SW	MH018082/2017/Claraval/M	.	.	.	.	.	.	.	.	.	.	.	.	.	.	.	.	.	.	.	.	.	.	.	.	.	.	.	.	.	.	.	.	.	.	.	.	.	.	.	.	.	.	.	.	.
	BR-MG-SW	MF170971/2017/SaoRoquedeMinas/M	.	.	.	.	.	.	.	.	.	.	.	.	.	.	.	.	.	.	.	.	.	.	.	.	.	.	.	.	.	.	.	.	.	.	.	.	.	.	.	.	.	.	.	.	.
	BR-MG-SW	MH484430/2017/Sacramento/M	.	.	.	.	.	.	.	.	.	.	.	.	.	.	.	.	.	.	.	.	.	.	.	.	.	.	.	.	.	.	.	.	.	.	.	.	.	.	.	.	.	.	.	.	.
	BR-GO	MK728873/2017/GO21/Amorinópolis /H	.	.	.	.	.	.	.	.	.	.	.	.	.	.	.	.	.	.	.	.	.	.	.	.	.	.	.	.	V	.	.	.	.	.	.	.	.	.	.	.	.	.	.	.	.
	BR-GO	MK333804/2015/GO27/Novo Brasil/M	.	.	.	.	.	.	.	.	.	.	.	.	.	.	.	.	.	.	.	.	.	.	.	.	.	.	.	.	.	.	.	.	.	.	.	.	.	.	.	.	.	.	.	.	.
	BR-RS	KY861728/2008/Caibate/M	.	.	.	.	.	.	.	.	.	.	.	.	.	K	K	.	.	.	.	V	.	.	G	.	N	.	.	.	V	.	.	.	N	.	.	.	.	.	.	N	.	.	.	.	.
	BR-GO	MK333803/GO05/2017/Nova Crixás/M	.	.	.	.	.	.	.	.	.	.	.	.	I	.	K	.	V	.	.	V	.	.	G	.	N	.	.	V	V	.	.	.	N	.	.	.	.	.	.	.	.	.	.	.	.
	BR-RR	JF912190/BeH655417/2002/Alto Alegre/H	.	.	.	.	.	.	.	.	.	.	.	.	.	.	K	.	.	.	.	V	.	.	G	.	N	.	.	.	V	.	.	.	N	.	.	.	.	.	.	.	.	.	.	.	.
	Ven	KM388817/Venezuela/2A/2004/M	.	.	.	.	.	.	.	.	.	.	.	.	.	.	K	K	.	.	.	V	.	.	G	.	N	.	.	.	V	.	.	.	N	.	.	.	.	.	.	.	.	.	.	.	.
	Ven	KM388814/Venezuela/6A/2005/H	.	.	.	.	.	.	.	.	.	.	.	I	.	.	K	.	.	.	.	V	.	.	G	.	N	.	.	.	V	.	.	.	N	.	.	.	.	.	.	.	.	.	.	.	.
	Ven	KM388818/Venezuela/8A/2006/M	.	.	.	.	L	.	.	S	.	.	N	.	.	.	K	.	.	.	.	V	.	.	G	.	.	.	.	.	V	.	.	.	N	.	.	.	.	.	.	.	.	.	.	.	.
	Ven	KM388815/Venezuela/9A/2007/M	.	.	.	.	.	.	.	S	.	.	.	.	.	.	K	.	.	.	.	V	.	.	G	.	N	.	.	.	V	.	.	.	N	.	.	.	.	.	.	.	.	.	.	.	.
	Ven	KM388816/Venezuela/10A/2010/M	.	.	.	.	.	.	.	.	.	.	.	.	.	.	K	.	.	.	.	V	.	.	G	.	N	.	.	T	V	.	T	.	N	.	.	.	.	.	.	.	.	.	.	.	.
TT	TT	HM582851/Trinidad and Tobago/TVP11767/2009/M	.	.	.	.	.	.	S	.	.	.	.	.	.	K	K	.	.	.	.	V	.	.	G	A	N	.	.	.	V	.	.	.	N	.	.	A	.	.	.	.	.	.	Q	.	.
1D	BR-MG	MG969501/2001/Bom Despacho/H	.	.	.	.	.	.	.	.	.	F	.	.	.	.	K	.	.	.	P	V	V	I	G	A	D	.	.	.	V	.	.	.	N	.	.	.	R	.	.	.	.	.	.	.	.
	BR-RS	JF912189/2001/S Miguel Missões/V	.	.	.	.	.	.	.	.	.	.	.	.	.	.	K	.	.	.	.	V	.	.	G	A	D	.	.	.	V	.	.	.	N	.	.	.	R	.	.	.	.	.	.	.	.
	BR-GO	JF912188/2000/AltoParaiso/H	.	.	.	.	.	.	.	.	.	.	.	.	.	.	K	.	.	.	.	V	.	.	G	A	D	.	.	.	V	.	.	.	N	.	.	.	R	.	.	.	.	.	.	.	.
	BR-GO	JF912187/2000/Goias/H	.	.	.	.	.	.	.	.	.	.	.	.	.	.	K	.	.	.	.	V	.	.	G	A	D	.	.	.	V	.	.	.	N	.	.	.	R	.	.	.	.	.	.	.	.
1C	BR-MG	JF912186/1994/ Arinos/H	F	.	.	.	.	.	.	.	.	.	.	.	.	K	K	.	.	I	.	V	.	.	G	.	N	.	.	.	V	.	.	.	N	.	.	.	.	.	R	.	V	.	.	.	.
	BR-PA	JF912184/1987/ Breves/H	F	.	.	.	.	.	.	.	.	.	N	.	.	K	K	.	.	I	.	V	.	.	G	.	N	.	.	.	V	R	.	.	N	.	.	.	.	.	R	.	.	.	.	.	.
	BR-PA	JF912183/1984/ S.Domingos do Capim/H	F	.	.	.	.	.	.	.	.	.	N	.	.	.	K	.	.	I	.	V	.	.	G	.	N	R	.	.	V	.	.	.	N	.	.	.	.	.	R	.	.	.	.	.	.
	BR-GO	JF912179/ 1980/Uruaçu/V	F	.	.	.	.	.	.	.	.	.	N	.	.	K	K	.	.	I	.	V	.	.	G	.	N	.	.	.	V	R	.	.	N	.	.	.	.	.	R	.	.	.	.	.	.
1B	BR-MS	JF912185/BR-MS/ BeAR513008/1992/Sidrolândia/V	.	.	T	.	.	.	.	.	.	.	.	.	.	.	K	.	.	.	.	V	.	.	G	A	N	.	M	.	V	.	.	.	N	V	I	.	.	.	.	.	.	.	.	F	.
	BR-PA	JF912182/1984/Monte Alegre/H	.	.	.	.	.	.	.	.	.	.	.	.	.	.	K	.	.	.	.	V	.	.	G	A	N	.	.	.	V	.	.	.	N	.	.	.	.	.	.	.	.	I	.	.	.

2016-2018 amino acid signature

Original pre-epidemic motif (1E clade

Polymorphism specific to MG/ES/RJ group
