# Supplementary material for: Distinct YFV Lineages Co-circulated in the Central-Western and Southeastern Brazilian Regions From 2015 to 2018
Source: Front Microbiol. 2019 May 24;10:1079. doi: 10.3389/fmicb.2019.01079 (PMC6543907; doi:10.3389/fmicb.2019.01079)
Supplement: Supplementary file 5 [file Data_Sheet_5.PDF]

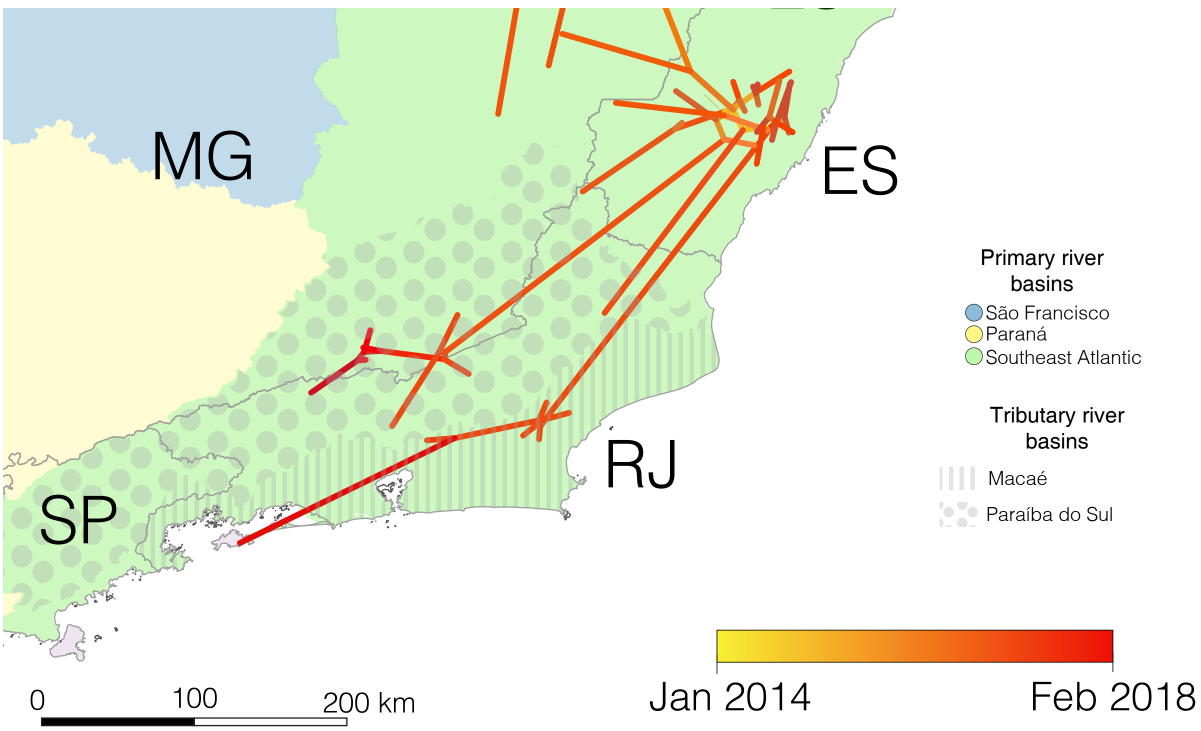

Supplementary Figure 4. Close view of the reconstructed spatiotemporal diffusion of the $YFV_{2015\text{-}2018}$ lineage in the primary Southeast Atlantic basin and its tributary river basins. Phylogeny branches were arranged in space according the internal nodes locations inferred by the continuous phylogeographic model. Branches were colored according to time as indicated by the legend. The gray lines represent the Brazilian states boundaries. The colored areas represent the different primary river basins while the gray patterns indicate the tributary river basins, according with the legend; additional details on tributary basins are available from IBGE (2019). Brazilian states: ES, Espírito Santo; MG, Minas Gerais; RJ, Rio de Janeiro; SP, São Paulo.
